# The Feasibility and Usability of RunningCoach: A Remote Coaching System for Long-Distance Runners †

**DOI:** 10.3390/s18010175

**Published:** 2018-01-10

**Authors:** Daniel Aranki, Gao Xian Peh, Gregorij Kurillo, Ruzena Bajcsy

**Affiliations:** Department of Electrical Engineering and Computer Sciences, UC Berkeley, Berkeley, CA 94720, USA; pehgaoxian@berkeley.edu (G.X.P.); gregorij@eecs.berkeley.edu (G.K.); bajcsy@eecs.berkeley.edu (R.B.)

**Keywords:** remote coaching, telemonitoring, telehealth, cadence, marathon, elevation change analysis

## Abstract

Studies have shown that about half of the injuries sustained during long-distance running involve the knee. Cadence (steps per minute) has been identified as a factor that is strongly associated with these running-related injuries, making it a worthwhile candidate for further study. As such, it is critical for long-distance runners to minimize their risk of injury by running at an appropriate running cadence. In this paper, we present the results of a study on the feasibility and usability of RunningCoach, a mobile health (mHealth) system that remotely monitors running cadence levels of runners in a continuous fashion, among other variables, and provides immediate feedback to runners in an effort to help them optimize their running cadence.

## 1. Introduction

Researchers have found that up to 79% of long-distance runners are expected to sustain a running-related injury in the lower extremities [1]. Such injuries could potentially be avoided if the long-distance runner runs within the boundaries of the recommended cadence throughout the entire run. Findings have shown that optimal control over one’s cadence can aid the runner in reducing the impact forces on joints [2], reducing muscle soreness and fatigue [3] and increasing efficiency of oxygen use [4]; all of which reduce the possibility of injuries to the runner. Given the numerous advantages of maintaining an optimal cadence throughout a run for long-distance runners, we are interested in examining a mobile health (mHealth) solution to monitoring and coaching cadence for long-distance runners, aimed at minimizing their risk of injury.

There are many commercial apps that are widely used by runners, including MapMyFitness, Runtastic, Adidas miCoach, Nike+, RunKeeper and Endomondo [5,6,7,8,9,10]. The primary function of most of these apps is to monitor the runner’s performance and to provide an interface for the runner to view statistics related to her or his runs. Some of these apps allow the runner to import workout plans that are aimed at motivating the runner and improving her or his performance. These apps, however, do not address minimizing the risk of injury, which creates a gap in the technology that we attempt to fill in this work.

To close this gap, we have designed an Android smartphone app, RunningCoach, to coach long-distance runners. In this work, we define “long-distance runners” as individuals who every week: (i) run for at least five kilometers (or three miles) in distance; or (ii) run at least one session that is one hour or longer in duration; which is the definition of the Association of Athletics Federations (IAAF). RunningCoach is designed to monitor the runner’s cadence, among other parameters, and to coach her or him based on the collected data. In this paper, we discuss the design and findings of a pilot study that aimed to explore the feasibility and usability of this app. This is the first of a series of studies that aim to refine the system and validate its efficacy regarding the reduction of injuries.

This paper extends on previous published work in [11]. Our previous paper was focused on the design and implementation details of the system. In this paper, we describe a feasibility and usability study for RunningCoach and report the findings of this study. Distinctly from the previously published paper, the contributions of this paper are (i) presenting evidence for the feasibility and usability of a coaching system based on remote monitoring for long-distance running; and (ii) reporting on lessons learned that developers can rely on to build robust mobile coaching solutions for fitness and health applications.

Concretely, we aim to understand the following factors related to the use of RunningCoach. First, we study the battery consumption incurred by the app as a usability factor. Second, this work examines general usability-related scenarios that are related to the robustness of the system, such as its ability to recover from faults (e.g., server is down, lost internet connection, etc.). Third, we examine the accuracy of the system in estimating the runner’s cadence, speed, and other variables, as perceived by the runner. Fourth, we examine the privacy-related aspects of using this app and study the acceptability of the users to this technology through a post-study questionnaire. Finally, we explore a possible analysis relating cadence, speed and the gradient of elevation (in the path of the run) as a potential way to assess injury-related performance, which is a gateway to future directions of this research effort.

The rest of the paper is organized as follows. In Section 2, we survey the literature on related research. Subsequently in Section 3, we describe the study objectives and the study protocol. In Section 4, we give a brief summary of the architecture and implementation of RunningCoach before presenting and discussing the results of the study in Section 5. We finally close the paper in Section 6 by reflecting on our conclusions and directions of future research.

## 2. Related Work

Injuries stemming from long-distance running are studied extensively in the literature. In addition, there are many commercial products available in the market that aim to assist runners to avoid injuries and to provide motivational support to lead a healthier lifestyle. These commercial products include variety of smartphone apps, often paired with wearables or insoles, that measure fitness markers such as energy expenditure, speed, distance, heart rate, and cadence (see the review of related fitness trackers in [12] and apps in [11,13,14]).

In this section, we focus on reviewing scientific literature on (i) studies that utilize remote monitoring systems for runners, (ii) studies that link running cadence to running-related injuries and (iii) studies that explore the usability and feasibility of mHealth systems in the context of running.

### 2.1. Monitoring Systems for Runners

Researchers have studied the effectiveness of various features present in remote monitoring systems for runners. Boratto et al.  studied the effectiveness of u4fit, a human-in-the-loop remote monitoring system linking runners with professional fitness coaches to enhance runner safety and engagement [15]. Similarly, Vos et al.  describe the design process and evaluation of Inspirun, a smartphone application for recreational runners [16]. Both papers emphasize the importance of personalized running experience and coaching. Both u4fit and Inspirun track heart rate, running speed, and GPS coordinates to keep track of the runs and to determined the intensity of the training. Given an intensity profile and training results, u4fit relies on a human fitness coach to provide feedback on compliance with the personalized training regimen. On the other hand, Inspirun relies on the smartphone application itself to provide coaching feedback. u4fit is aimed at improving and sustaining runner motivation, while Inspirun is designed to help recreational runners set new performance-related goals. The authors, however, do not report on any quantitative results in their publications. Unlike u4fit or Inspirun, our efforts are focused on minimizing injuries in a specific subpopulation of runners, i.e., long-distance runners. Moreover, in RunningCoach, the training regimen is focused on cadence, which has been reported as one of the factors associated with injury and performance.

Our long-term goal, which we aim to achieve after a series of further studies, is to develop a recommendation algorithm that outputs an optimal cadence level for an individual runner, based on her or his physical parameters (e.g., age, gender, height and weight), data from previous runs (e.g.,  performance, heart rate, etc.) and other factors related to injuries.

### 2.2. Taxonomy of Running Cadence

Up to 79% of long-distance runners are expected to sustain a running-related injury within a six month period, most commonly located at the knee [1,17]. Patellofemoral pain syndrome (PFPS) was observed as the most frequent issue encountered in running injuries out of the 26 most common running injuries [18]. PFPS is a condition that causes severe to mild knee pain, which often starts due to a dramatic change in the training regimen [19]. In addition to this reason, numerous biomechanical risk factors can contribute to causing PFPS. These factors include kinematic abnormalities, patellar maltracking, overuse, and excessive compressive stresses on the patellofemoral joint cartilage [20,21,22,23,24]. Running as an activity generates much larger cartilage stress at the patellofemoral joint as compared to other everyday activities [25,26]. A clear way forward to mitigate PFPS would be to find a method to reduce the magnitude of the patellofemoral joint force during running. By running at 5% to 10% above one’s preferred cadence has been shown to be beneficial in reducing pain, increasing training ability in runners with PFPS and minimizing the risk of injury to the patellofemoral joint. For instance, Lenhart et al.  report a decrease of 14% in peak patellofemoral joint force as a result of a 10% increase in cadence relative to the preferred cadence level [27]. Heiderscheit et al.  report a significant decrease in the absorbed mechanical energy at the knee as a result of 5% to 10% increase in cadence relative to the runner’s preferred cadence level [2]. These reductions in peak patellofemoral joint force and absorbed mechanical energy may reduce the risk of running-related knee injuries [2,27].

The importance of optimizing one’s cadence as a long-distance runner is twofold. First, subtly increasing and optimizing cadence during a run assists runners with preventing common running-related injuries [2]. From an injury-prevention standpoint, this enables both professional and hobbyist long-distance runners to continue running. Second, optimizing and maintaining a consistent cadence throughout a run enables long-distance runners to conserve energy and as such enhance performance [28,29]. Unlike novice long-distance runners, advanced long-distance runners avoid deviations from their optimal cadence when they are in a fatigued state as such deviations lead to an increased energy cost [30,31,32].

Given the numerous benefits of optimizing cadence for long-distance runners, many mHealth technologies were created to meet this need. In order to provide effective feedback on running cadence, many have studied the relationship between music and setting the running cadence through auditory-motor synchronization [33,34]. These studies provide evidence of the ability of music to affect the running cadence and ultimately improve performance [33] and reduce injuries [2,34].

### 2.3. Feasibility and Usability of mHealth Systems

In spite of findings that show that a lack of focus on usability and feasibility issues for mHealth systems would lead to an increase in overall costs and delays in successful implementation, few mHealth interventions have explained the attributes that contribute to their success and the aspects that have led to failed implementations [35,36,37]. A 2013 systematic review of mHealth literature by Fiordelli et al.  identified that only 14% of the studies reported on user assessment of the technology [38].

Through post-study questionnaires in the first user study, Vos et al.  managed to surface issues with Inspirun on the accuracy of speed measurements which were later addressed in the third release of Inspirun [16].

In mHealth, understanding the privacy preferences of users and their acceptability of the technology is a determining factor in its success and adoption due to the amount of sensitive data typically collected by the apps. Very few studies, however, report on the privacy-related aspects of the mHealth systems they adopt [13,39].

## 3. Materials and Methods

### 3.1. Study Objectives

The short-term objective of the study is to assess the feasibility and usability of RunningCoach, a mobile health (mHealth) remote coaching system for long-distance runners which aims to optimize their running cadence. We are particularly interested in understanding (i) how long-distance runners interact with RunningCoach; (ii) how long-distance runners perceive the accuracy of the data collected by RunningCoach; and (iii) what running-related analyses can be performed with the collected data to provide further insights into the system and runner’s performance. These immediate objectives are set for the purpose of guiding the future iterations of the study. Understanding the interactions of the user with the system, and investigating any potential usability issues with the system allow us to address those issues in the future. Moreover, exploring potential running-related analyses helps us devise hypotheses, that can be validated in future studies.

The long-term objective of our research is to achieve personalized coaching for an individual runner that will be integrated in the proposed telemonitoring system. We envision this coaching system to include the ability to take advantage of the anthropometric parameters of each individual, the individual’s previous performance, and other factors related to injuries in order to devise a training regimen that is tailored to that individual. Ultimately, this personalized training regimen shall provide recommendations to the runner regarding cadence and speed, depending on the specific consecutive day of training and the previously-collected running data.

In order to achieve these objectives, we have designed a series of user studies, which are approved by the Institutional Review Board at University of California, Berkeley. In this paper, we describe the design and findings of the first study in this series, which is concerned with the feasibility and acceptability of such telemonitoring technology.

### 3.2. Training Regimen

**Figure 1:**
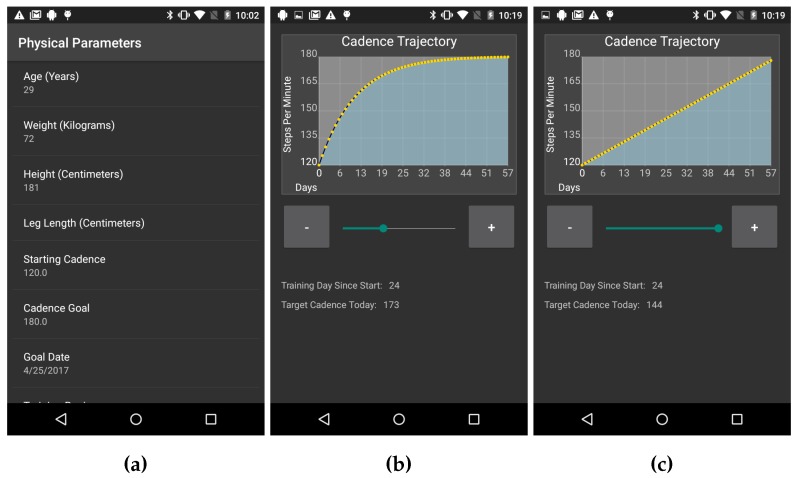
(**a**) A screenshot depicting the physique profile screen; (**b**) a screenshot depicting an exponential cadence training regimen; and (**c**) a screenshot depicting a linear training regimen [11].

In order to guide the runners to improve their running cadence, the system has to provide a training regimen tailored to each subject. Before establishing the plan for improving the runner’s cadence, the system has to establish the runner’s baseline cadence. Note that the different runners may have different levels of experience and different body types. As such, a single and fixed training regimen may not be generalizable to all runners. Therefore, RunningCoach collects two types of information from the runner in order to set her or his personalized training regimen. First, RunningCoach collects information about the runner’s physical parameters. The collected physical parameters in the app include age, gender, height, weight, and leg length as measured from the hip joint to the ground (Figure 1a). Second, RunningCoach sets the desired cadence improvement curve, by collecting information about the runner’s baseline, target cadence and the length of the proposed training regimen. In the current version of RunningCoach, all of the aforementioned parameters are manually set by the runner.

In future iterations of the app, a recommended training regimen will be determined by collecting data over a small set of consecutive runs and comparing runner’s own baseline with similar runners. Similar runners will be identified using the provided physical parameters and their baseline data.

After the runner provides her or his physical parameters, a default training regimen is suggested. This training regimen consists of a starting cadence level (baseline), a target cadence level, the length of the training regimen, and the steepness of the cadence improvement curve. The default length of the training regimen is 90 days, which can be altered by the subject. The reason for selecting 90 days as a default value was to maintain the length of the training regimen with the length of the study. The family of parametric cadence training regimens adopted by RunningCoach follows an exponential improvement curve, as follows.

(1) $$C\left(d\right)=\frac{C_{N}\cdot e^{\alpha N}-C_{0}}{e^{\alpha N}-1}-\frac{C_{0}-C_{N}}{e^{-\alpha N}-1}\cdot e^{-\alpha d},$$

In Equation (1), C(d) denotes the suggested cadence on day *d*. $C_{0}$ denotes the baseline cadence of the runner, where $C_{N}$ denotes the target cadence. The parameter α controls the steepness of the personalized training regimen (larger values imply steeper improvements) and *N* denotes the length of the training regimen in days. By setting values for $C_{0}$, *N*, $C_{N}$ and α, a training regimen is established that guides the runner to achieve the target cadence level $C_{N}$ within *N* days.

The family of training regimens described in Equation (1) is the solution of the function $C\left(d\right)=A+B\cdot e^{-\alpha d}$ with initial conditions $C\left(0\right)=C_{0}$ and $C\left(N\right)=C_{N}$. Moreover, as α approaches 0, the training regimen described in Equation (1) approaches a training regimen with a linear improvement curve. Concretely, $\lim_{\alpha \to 0}C\left(d\right)=C_{0}+\frac{C_{N}-C_{0}}{N}\cdot d$. This claim is formally shown in [40]. The reasoning behind devising training regimens with gradual improvements in cadence is to minimize the risk of injury due to sudden changes in the runner’s training routine. In addition, the exponential training regimen allows for larger increases around the baseline and then levels off towards the higher target cadence to prevent over-training.

Examples of the training regimens are depicted in Figure 1b,c, where Figure 1b shows a training regimen with exponential improvements in cadence (α>0) and Figure 1c shows a training regimen with a linear improvement curve of cadence (α→0).

As stated earlier, in the current version of RunningCoach, the cadence training regimen settings are manually set by the runner. Eventually, we aim to develop an algorithm that would recommend, to each runner, her or his ideal cadence level ($C_{N}$), and a personalized improvement steepness curve (α), that are based on her or his physique profile depicted in Figure 1a as well as data from her or his previous runs. In addition, we aim to use heart rate data to dynamically alter the training regimen for the runner in a way that is sensitive to the runner’s physical ability. In order to achieve this goal, we aim to use the data collected in this study (and the future iterations of this study) to train a recommendation algorithm in a way that mimics the true improvement trajectories of the runners. Further studies are needed to validate the efficacy of such a recommendation algorithm. More details about the adopted training regimen can be found in [11,40].

### 3.3. Study Protocol

#### 3.3.1. Screening and Recruitment

Members of the University of California Berkeley community were sought for participation in this study. Potential participants were screened according to two criteria. First, participation was only allowed to those who are current long-distance runners. This requirement was placed in an effort to not subject the study participants to the risks of running, if they do not regularly run (e.g., injury). By limiting participation in the study to those who regularly run long distances, we are taking measures to minimize the risks of participation in the study. For screening purposes, we use the definition of “long-distance runner” that is used by the International Association of Athletics Federations (IAAF), which states the following:

A long-distance runner is someone who every week (i) runs for at least 5 km (or 3 miles) in distance; or (ii) runs at least one session that is 1 h or longer in duration.

The second screening criterion limited participation to subjects who owned an Android smartphone running Android 4.3 (Jelly Bean MR2) or newer. This requirement allowed us to better assess acceptability and usability qualities of the proposed system, by requesting that participants use their own smartphones for the purposes of the study. In turn, any feasibility or usability issues arising from using an unfamiliar smartphone, if it were to be provided by the study, are thus eliminated.

After the screening of each candidate subject, the researcher administering the process obtained her or his consent for participation in the study. After the consent process, the researcher conducted the initial set up procedure for the subject. This procedure entailed providing the subject with a Jarv Run heart rate chest strap monitor [41]. Afterwards, the RunningCoach app was installed on the subject’s smartphone and paired with the heart rate monitor. Subsequently, the subjects were instructed on the proper way of using the system by providing instructions specific to pre-, during, and post-running use (e.g., how to wear the heart rate chest strap, where to secure the phone during the run, etc.). Finally, the researcher demonstrated the use of the RunningCoach app and its features to the subject. The subjects were encouraged to ask any questions related to the system or the protocol.

In total, six subjects were recruited for the study. The study spanned from February 2017 to July 2017. The subject demographics and physical parameters are summarized in Table 1.

#### 3.3.2. Study Procedures

During the main part of the study, the subjects were asked to secure the phone on their body in a comfortable area (e.g., on the shoulder or on the hip) during their routine runs with the RunningCoach app. In addition, the subjects were asked to wear the heart rate chest strap that was provided to them, which connects to the app via Bluetooth and sends the data in real time. The subjects were not specifically asked to use the app during each run but rather to use it on their own terms. The reason subjects were not instructed to use the app during each run is that some recruited subjects are competitive runners who preferred to use a smartphone only during a part of their weekly training routine.

During each run, the app collects information about the runners’ estimated energy expenditure, cadence, speed, heart rate from the chest strap, and total distance covered. In addition to these estimates, the app collects the following two variables: (i) whether or not the screen light is on; and (ii) the battery level. Before and after each run, the app collects single estimates of the heart rate using two different vision algorithms, one from a video of the subject’s face and another from a video of the subject’s index finger [11]. These algorithms were previously only validated under controlled conditions. Since an external heart monitor was used in the study, we used this opportunity to get an insight on the usability of the implemented heart rate measurement algorithms in the field. Note that this was not a controlled validation and should therefore be treated as exploratory only. Table 2 summarizes the types of data collected before, during and after the run.

**Figure 2:**
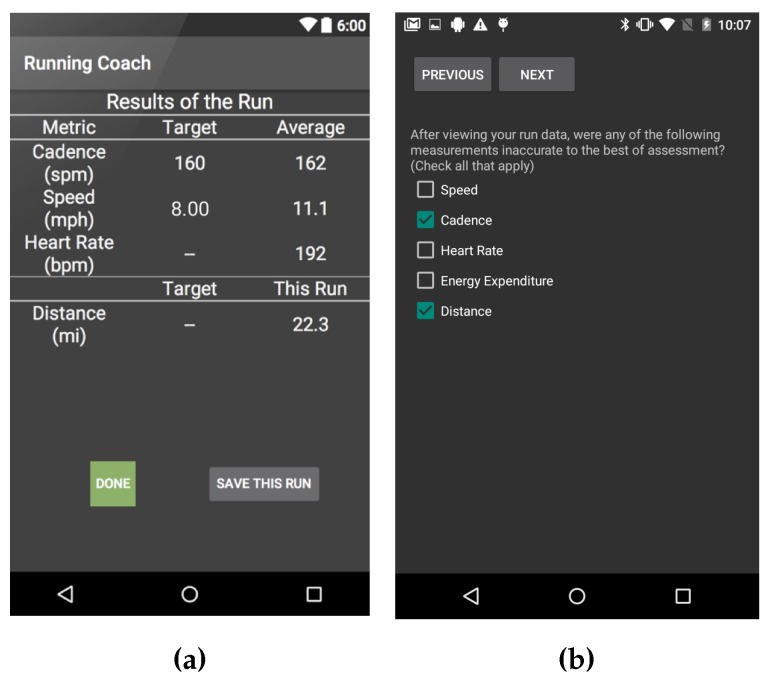
(**a**) A screen showing some of the run-statistics after a run; and (**b**) an example of a question in the post-run survey [11].

After each run, subjects were asked to fill a post-run survey inquiring about the run. The questions asked in the post-run survey were: (i) “How tired were you on a scale of 1-5 where 3 is your typical level of fatigue after long runs prior to using the app, 5 is very tired and 1 is least tired?” (ii) “After viewing your run data, were any of the measurements inaccurate to the best of your assessment? (Choose all that apply from Speed, Cadence, Heart Rate, Energy Expenditure, Distance);” (iii) “If you selected any of the choices in the previous question, please explain;” and (iv) “Please provide any other comments regarding your experience using the app.” Figure 2a depicts an example screen showing some of the run statistics after the run, and Figure 2b depicts an example of a question from the post-run survey as displayed in the app. The post-run survey provides information about (i) the perceived accuracy of the system; (ii) the usability of the system; and (iii) how hard the training regimen is pushing the runners in terms of performance. The post-run questions about the perceived accuracy of the app’s collected data are shown to the subject after the run statistics are presented (e.g., Figure 2a).

In the process of designing the post-run survey, the 1 to 5 scale of fatigue was selected for the following reasons. The adopted scale is a reduced version of rating-of-fatigue (ROF), which is a 10-point scale designed to measure level of fatigue [42]. Other seemingly relevant measures have been studied in the sports literature, including Borg’s perceived exertion scale [43]. Borg’s scale is not a good fit for our purposes because it is designed to capture subjective exertion. Some researchers argue that perceived exertion, the subjective experience of how hard a physical task feels, is different from perceived fatigue and should not be used to measure perceived fatigue levels [42]. Moreover, Borg’s scale is designed to follow the heart rate of the subject by multiplying it by 10. Since we are collecting heart rate data, Borg’s score would not provide additional information, and therefore, a reduced fatigue scale that is similar to ROF was selected for our purposes.

Subjects were asked to perform the aforementioned procedure for a period of 3 months. By the end of that period, the subjects answered an exit acceptability and privacy survey about the system and the study.

## 4. System Design

The architecture and implementation of the system, which are based on the Berkeley Telemonitoring framework [44], are described in [11]. For completeness, we briefly discuss them in this section; for more details, we refer the reader to [11,39,44].

### 4.1. RunningCoach App

**Figure 3:**
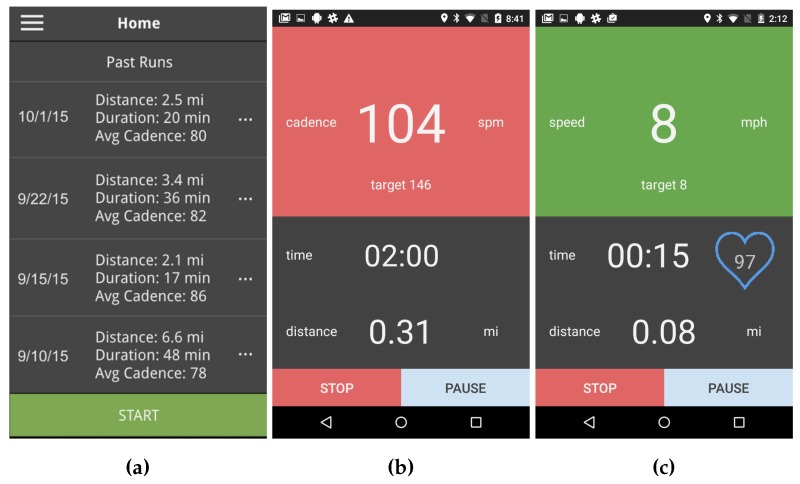
(**a**) A screenshot of the home screen of RunningCoach, summarizing the past runs; (**b**) a screenshot from the app during a run showing the cadence significantly lower than its target value (outside the 10% range of the target cadence); and (**c**) a screenshot from the app during the run showing the speed within acceptable range of its target value (within 10% of the target speed) [11].

The purpose of the RunningCoach app is to serve as the remote monitoring node. As such, it collects data about each run, including energy expenditure, cadence, speed, heart rate, and distance covered. In addition, the app administers the surveys after each run. Figure 3 depicts various screens from the RunningCoach app. Concretely, Figure 3a depicts the home screen of the app, listing previous runs; Figure 3b,c depict two screens shown during the run, presenting the runner’s cadence and speed, respectively.

Finally, the app delivers the real-time feedback to the runners regarding their cadence and/or speed levels (depending on the settings). If the cadence or speed are outside of a preset range around the target values of the day, according to the training regimen, the phone provides haptic feedback (vibration) and auditory cues (beeping) to the runner. The vibration and beeping patterns depend on whether the runner is higher than the target value or lower than it, allowing the runner to adjust accordingly. This preset range can be set by the subject in the app, with a default value of 10% (around the target cadence or speed).

### 4.2. Backend

The RunningCoach server backend is written in Java and uses the Berkeley Telemonitoring framework as well. To communicate with the client nodes, the backend uses the Tele-Interfacing (TI) protocol with Transport Layer Security (TLS), as described in [44]. The backend receives the data from the client nodes in the form of data jobs, unpacks the jobs back into encapsulators using job handlers (conforming to the Berkeley Telemonitoring framework) and stores the data in a MySQL database. Each data job is attached to a subject identifier that is uniquely set to each runner in the app. The identifiers are used to identify the source of the data (Table 1). The subject identifier is stored in the database along with the corresponding data.

### 4.3. Dashboard

**Figure 4:**
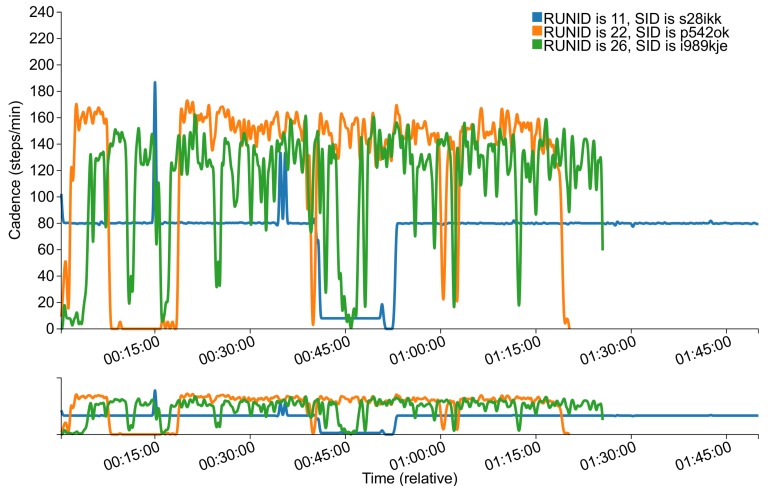
An example plot from the dashboard, depicting the estimated cadence during runs 11, 22 and 26 by subjects s28ikk, p542ok and i989kje, respectively [11].

In addition to the backend, we designed a dashboard that provides a way to visualize the data about the runs. The dashboard can be accessed on the web using a browser. Figure 4 depicts an example plot of cadence data for three different runs by three different subjects. In the runs reported in Figure 4, runner s28ikk reported holding the phone in his hand during run 11, contrary to the instructions given to the subjects to place the smartphone around the hip or on the shoulder during the run. In all of the runs in the figure, we observe that the runners stopped momentarily, which is corroborated by the other data variables (e.g., speed, GPS, etc.). This explains the seemingly low cadence readings in these figures. These issues will be discussed further in Section 5.

**Figure 5:**
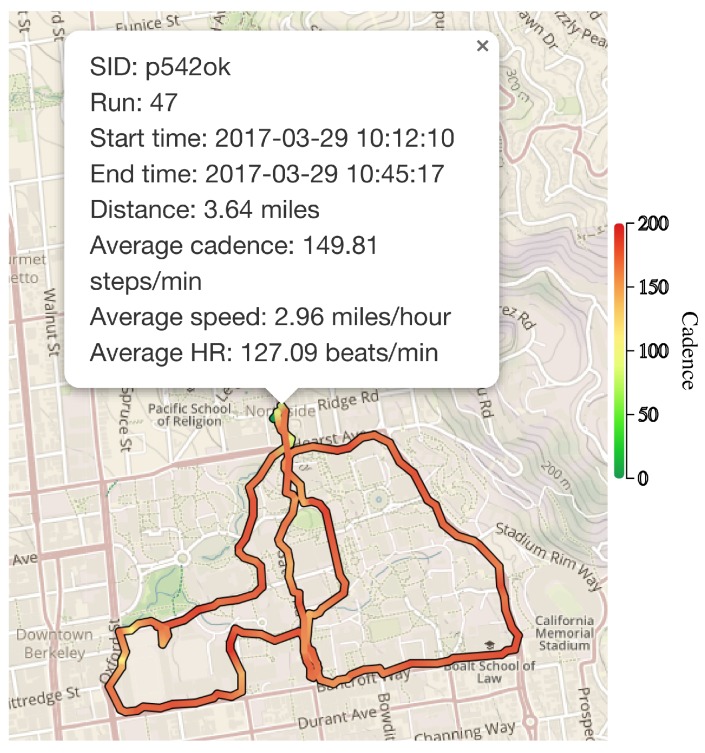
An example plot of the path of run 47 by subject p542ok.

The paths of the runs can also be visualized with a colored overlay, representing the recorded values from various data sources, such as cadence, speed, or heart-rate. For example, the dashboard can provide a plot of the path of the run with the color from green to red indicating the value of cadence (range: zero to 200), as depicted in Figure 5.

## 5. Results and Discussion

### 5.1. General Statistics

**Figure 6:**
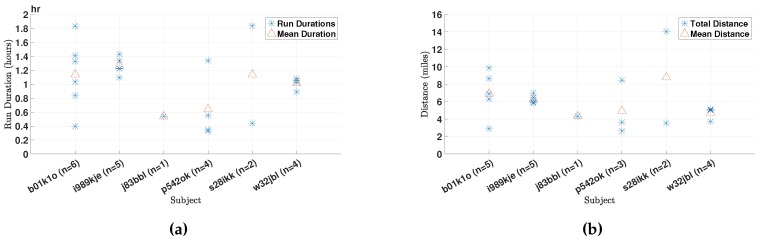
(**a**) The durations of the different runs as observed in each subject (N=22); and (**b**) the total distances of the different runs as observed in each subject (N=20).

**Figure 7:**
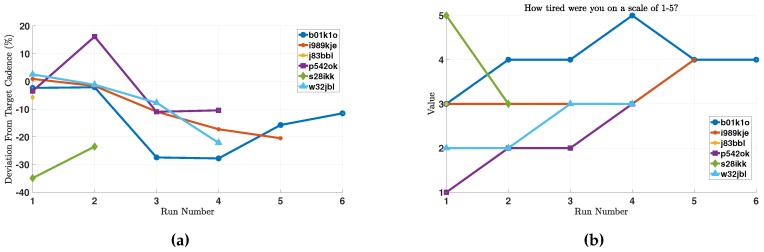
(**a**) The deviation of the average cadence from the target cadence for each run, expressed in percentage points (N=22); and (**b**) subject-reported level of fatigue after each run. A value of three represents the level of fatigue reported after an average run; a value of one is least fatigued; and value five is most fatigued after an average run (N=22).

The six subject enrolled in this study used the app to collect data of a total of 22 runs amounting to more than 22.5 h of data. In Figure 6a, we present the durations of the runs for the different subjects. In addition, Figure 6b depicts the total distances traveled during each run for the different subjects. Finally, Figure 7a presents the deviation of the runner from his or her target cadence for the run in question. Note that in some runs, the subjects elected to disable GPS data collection, and those runs were omitted from Figure 6b, which is why the number of runs per runner is different in the different plots.

We note that for the majority of runs, runners were running at a cadence lower than the recommended cadence for the run, signaling the need for more personalized training regimens as stated in our long-term goals. Moreover, we note that the auditory and haptic feedback is provided whenever the cadence is more than 10% off the target value for the run, for a period of 30 s or longer. Therefore, runs that had a deviation in cadence within a 10% window of the target cadence level should be considered as ones that met the target cadence. Moreover, runner s28ikk reported running with the phone in his hand. As will be detailed in Section 5.3.2, this violates the design assumptions of the cadence estimation algorithm, which explains the large deviation from the target cadence for that runner.

In order to provide contextual perspective, we present the self-reported levels of fatigue after each run by the different subjects in Figure 7b.

### 5.2. Usability

#### 5.2.1. Battery

**Figure 8:**
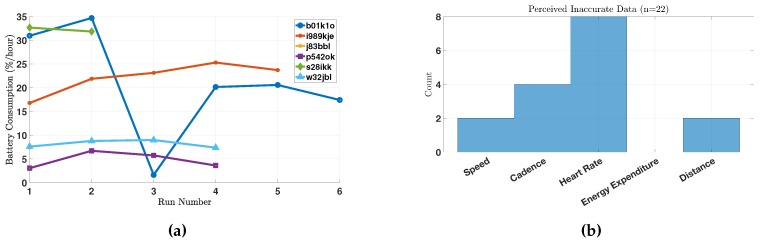
(**a**) The amount of battery consumption (in %) per hour during the different runs by the different subjects (N=22); and (**b**) the perceived accuracy of the collected data by the runners (N=22).

Optimizing battery consumption is key to adoption of smartphone-based telemonitoring applications [39,45]. In this study, the subjects did not report any usability issues regarding battery consumption. However, as described earlier, battery levels were collected during every run (once a minute). From these data, we calculate the amount of battery that is consumed during each run. Note that the battery consumption captures the total battery consumption of the smartphone, not just of our app. Since the different runs are different in duration, we normalize this consumption by time, in order to get a measure of “battery percentage consumed per hour”. These results are depicted in Figure 8a.

We note that different subjects have different battery consumption profiles, which may be caused by one or a combination of the following factors. First, different phones have different battery consumption profiles; and the phones used in this study were not provided by us and therefore are heterogeneous. Second, battery consumption depends on factors that are external to RunningCoach, such as listening to music and whether the music is streamed or played locally. Third, battery consumption profiles depend on the carrier and the strength of cellular coverage [46]. However, the data provide evidence that RunningCoach alone is not very burdensome on battery and can consume as low as 5% battery/h. This is even true for subject b01k1o, who manifests high battery consumption patterns in general. In one run, RunningCoach consumed less than 5% battery/h, which can be explained by the factors listed above.

#### 5.2.2. General Usability

We now turn to general usability issues as reported by the subjects. First, there were two instances where extreme fault-tolerance was tested during the study. In one instance, a subject reported that the phone’s operating system malfunctioned in the middle of a run; but when the phone was restarted after the run, the app resumed its operation from the previous state. In this instance, the data before the crash were not lost, even though they were not submitted to the server prior to the crash.

In another instance, the server was down for a prolonged period of time, during which several runs were taken by the different subjects. Because of the built-in fault-tolerance mechanisms in the Berkeley Telemonitoring framework, no data were lost during the server downtime. The app was able to recover all the data and send them to the server once the server was back online. It is worth reporting that one subject uninstalled the app manually before the server was restarted; which caused the data not yet sent to the server to be lost. These incidents validate the fault-tolerance implementation described in [44].

Besides the aforementioned issues, we further summarize general usability reports made by the study subjects in Table 3.

### 5.3. Perceived Accuracy

As part of the routine post-run surveys, we inquired about the perceived levels of accuracy of the collected data. Concretely, we asked the subjects: “After viewing your run data, were any of the measurements inaccurate to the best of your assessment? (Choose all that apply from Speed, Cadence, Heart Rate, Energy Expenditure, Distance)”. The responses to this question are presented in Figure 8b. As follow-up to this question, the subjects were also asked to provide additional information when they thought any measurements were inaccurate.

Energy expenditure was not perceived as inaccurate by the runners. A possible explanation to this is that it is difficult for people to gauge their own energy expenditure during physical activity. For distance, one subject reported: “if you’re counting total miles traveled, then it should be closer to 5, but if you’re just counting miles run, that’s maybe accurate.” The measured distance (from GPS data) during this particular run was 3.72 miles, which reflects the total miles traveled. After carefully reviewing the data, it seems that the run monitoring was not started until the runner was in the middle of the run. We concluded this because the runner always took the same route, except in this run where the monitoring started from a middle point in that route (which accounts for the difference in mileage). In the following sections, we discuss in more detail the perceived accuracy for the heart rate and cadence measurements.

#### 5.3.1. Heart Rate

We note that the heart-rate measurements were deemed to be the least accurate in our system. According to the responses of the follow-up questions in the post-run survey, the subjects found the pre- and post-run single heart-rate measurements using the computer vision algorithms to be inaccurate. This discontent can be explained by the fact that these algorithms were mainly tested in a controlled lab environment, and failed to perform at the same level when taken in uncontrolled settings. For example, on different occasions, subject p542ok responded to the follow-up questions with “I am not at 70 bpm immediately after a run [(referring to the vision-based estimates)]”, “face and finger[-based estimates] are way off as usual”, (they were lower than 55 bpm) “finger[-based estimates] is still way too low after the run” and “finger heart rate after run was 45”. In the first instance reported above, the heart-rate chest strap did indeed record 70 bpm at the end of the run. In all other instances when the subject complained about the accuracy of the vision-based heart-rate estimates, the heart-rate chest strap recorded values that were significantly higher than the vision-based ones. No feedback was given regarding the accuracy of the chest heart-rate strap.

On one occasion, the same subject took video-based heart rate estimates without actually running. The subject did not report any perceived inaccuracy in the estimates, which were 61 bpm and 62 bpm for the finger-video-based and face-video-based estimates, respectively. Other subjects did not provide textual feedback regarding the accuracy of the heart-rate measurements.

#### 5.3.2. Cadence

The subjects’ responses to the follow-up question provided valuable insight as to why they perceived certain cadence measurements as inaccurate. These reasons are sometimes explained by improper use of the system. For example, as a follow-up to choosing “cadence” as inaccurate in the survey, one subject responded: “Cadence too low; possibly because I held phone in hand”. The cadence algorithm was designed to work around the hip area or on the shoulder, which explains why during this run the average cadence of the runner was as low as 72 steps/min.

**Figure 9:**
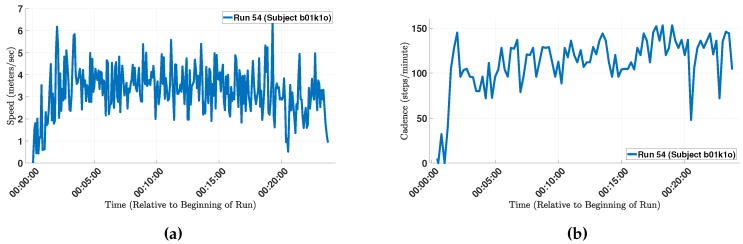
(**a**) The estimated speed (m/s) during run 54 by subject b01k1o; and (**b**) the estimated cadence (steps/min) during run 54 by subject b01k1o.

In another example, a subject reported that “My measured cadence was also lower than expected—I was mostly running on beat to songs that had 150+ bpm”. The measured cadence of the run is depicted in Figure 9b. The average cadence of the run was 111 steps/min, which includes the segments in the beginning of the run as well as some segments when the runner slowed down (or perhaps momentarily stopped running). After excluding these data points, the average increased to 124 steps/min, which is still lower than the subject’s self-reported cadence of 150 steps/min. For reference, Figure 9a depicts the speed during the same run, which shows that the runner would slow down in many instances. One potential reason for those momentary slow-downs is the urban path that the runner selected, which is corroborated by the run’s GPS data (the path passed through many street intersections).

### 5.4. Acceptability

**Figure 10:**
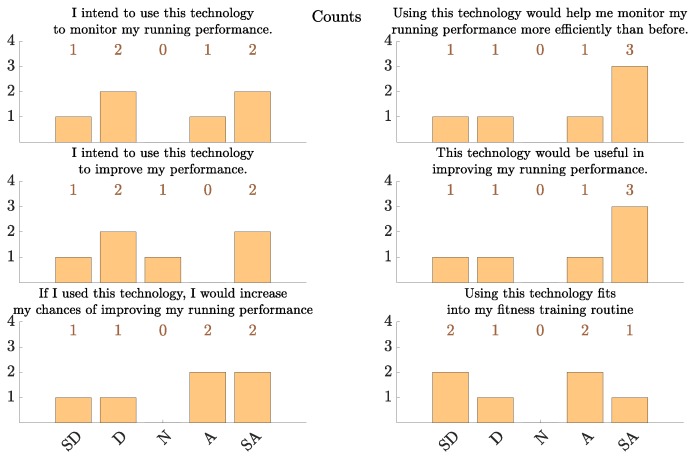
The results from the acceptability portion of the post-study questionnaire (N=6). SD = “strongly disagree”, D = “disagree”, N = “neutral”, A = “agree” and SA =“strongly agree” [47].

In Figure 10 we present the results of the acceptability portion of the post-study questionnaire. It is worth noting that the study subjects’ acceptability of this technology is lower than the acceptability reported in other telemonitoring applications such as in congestive heart failure (CHF) [46,47]. This can be attributed to multiple factors. First, some subjects voiced preference for using a monitoring device with a smaller form factor than a smartphone for this application. For example, one subject stated: “I’m a big fan of using running watches instead of phone apps because the form factor is much more comfortable. That’s the main reason I was so negative about using a phone-based athletic trainer”. In that regard, we note that the Berkeley Telemonitoring framework supports general Android devices, not only Android smartphones. Therefore, an Android smart watch application can be implemented using the same framework, averting the form factor challenge. Second, people may be more likely to tolerate certain drawbacks in a technology if they perceive a higher utility and value in it. As such, the higher levels of acceptability in CHF telemonitoring and intervention (e.g., [46]) may be attributed to the potentially higher perceived utility and value of the technology to the subjects in that application [47].

**Figure 11:**
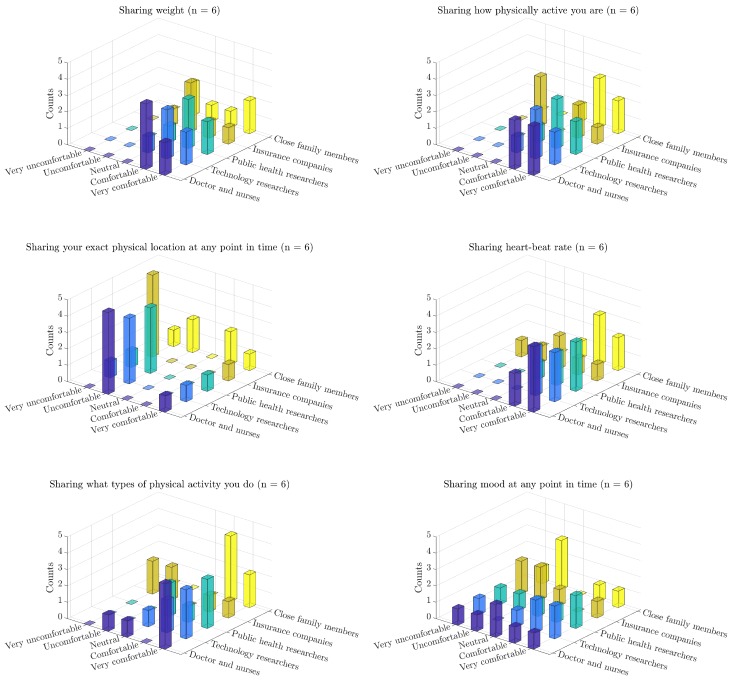
The responses to the privacy portion of the post-study questionnaire about the subjects’ comfort levels sharing different data variables with different parties (N=6) [47].

Another aspect of the post-study questionnaire focused on the privacy aspects of the technology, as perceived by the subjects. In that portion of the questionnaire, the subjects were asked the following set of questions. “Sometimes the smartphone might automatically record, or ask you to report, specific kinds of information about your health or behavior, such as your weight, your mood, or your blood pressure. The following questions will help us understand how comfortable you are with the idea of other people knowing these things about you”. In Figure 11 we present the subjects’ responses indicating their levels of comfort in sharing data about their (i) weight; (ii) level of physical activity; (iii) exact physical location at any point in time; (iv) heart-beat rate; (v) types of physical activity they do; and (vi) mood at any point in time, with: (i) doctors and nurses who provide them healthcare; (ii) researchers who study athletic training technology; (iii) public health professionals who study the effects of exercise and athleticism; (iv) insurance companies that set their health insurance prices; and (v) close family members who care about their health [47].

In particular, the data provide an indication that the subjects’ level of comfort in sharing data about their fitness, GPS, health and mood with technology researchers is comparable to sharing those variables with their family and physicians [47]. In contrast, the subjects were noticeably less comfortable sharing these variables with their health insurance companies, suggesting that they are not privacy indifferent. These two observations combined suggest that the provided technology is at an acceptable level from a privacy point of view. In addition to the ethical reasons for designing privacy-aware data-collection systems, these findings are of great significance because they have direct implications on the adoption of these systems [39].

We note that the privacy acceptability levels are similarly high to those reported in the other applications such as CHF [46,47].

### 5.5. Elevation, Cadence and Speed

It is interesting to understand whether runner’s speed or cadence change as a result in elevation changes during the run. That is, we ask whether cadence or speed drop as a result of running uphill and whether they increase as a result of running downhill, relative to the cadence and speed during running on a flat surface. This type of analysis can be beneficial in analyzing the performance of each runner for each run. In order to perform this analysis, we split each run into segments of one minute each. Each segment was defined as “running uphill” if the net change in elevation is at least +2 m. Each segment was defined as “running downhill” if the net change in elevation is at most −2 m. All other segments were marked as running on flat surface. In order to estimate elevation for each data point, we used the Digital Terrain Elevation Data (DTED) maps from the United States Geological Survey (USGS) [48] to estimate the elevation of each GPS data point during the run.

**Figure 12:**
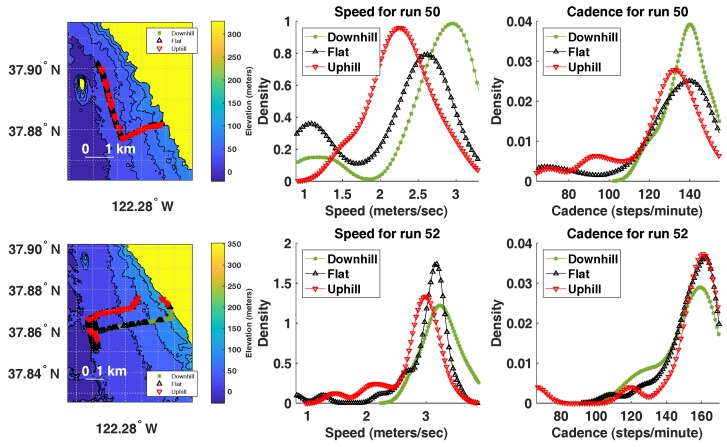
The relationship between elevation changes (ascending/descending), cadence and speed for runs 50 and 52 by subjects i989kje and b01k1o, respectively.

During each run and for each surface type (uphill, flat and downhill), we explored the distribution of cadence and speed. It is ideal if the speed and cadence remained unaffected due to terrain elevation changes. The data indicate that, for most runners, cadence is less affected by terrain elevation changes than speed. Figure 12 displays this analysis for two runs by two different runners. In run 50, there seems to be a noticeable change in speed due to terrain elevation changes; however, cadence is less affected. In run 52, there is a smaller effect of terrain elevation changes on both speed and cadence, indicating a more consistent pace during the run (which is desired). This type of running performance analysis is possible with the type of data collected by RunningCoach. One can envision this analysis as a source of intervention to help the runner minimize injuries, although further longitudinal studies are necessary to validate this claim.

### 5.6. Limitations of the Study

The presented study has several limitations. First, the number of subjects in the study (sample size) is small. While all recruited subjects are considered long-distance runners per definition, their training regimens in total distances traveled per run were quite diverse. Some subjects were in general not users of smartphone apps for running, therefore their expectations and responses may have been different than of the subjects who are users of such applications on a more regular basis. Since the goal of this study was to assess the feasibility and the usability of the developed telemonitoring framework, less emphasis was given on the rigid study protocol that would perhaps result in larger number of collected runs during the three months. The study instead aimed to investigate how often the runners would use RunningCoach app during their runs and what type of information can be extracted from the collected data. A sample of the results was presented in this paper. To evaluate in more detail the running performance of individual runners and their changes over time, given the feedback on the cadence, the subjects in future studies will be required to perform certain number of runs per week. Such data collection across larger pool of subjects would provide the data needed to achieve the long-term goal of this research, i.e., to tune the training models to individual runner’s physique and to evaluate the efficacy and potential benefits of such training for prevention of injuries.

Furthermore, feedback from the users indicated that the large form factor of the smartphone may not be as convenient for long-distance runners due to the mounting inconvenience and additional weight. The use of a smartphone for casual monitoring of daily activities may be preferred over wearable devices as people tend to carry their smartphone throughout the day. On the other hand, to measure the athletic performance, wearable sensors may be more convenient. As the Berkeley Telemonitoring framework is compatible with any Android device, the future iterations of this system may include the use of a smart watch or other wearable technology.

## 6. Conclusion

Studies suggest that optimizing cadence is an important factor in reducing the risk of sustaining a running-related injury and in improving overall running performance. In this work, we presented a feasibility study utilizing an mHealth solution to long-distance running cadence-based coaching, called RunningCoach. Future versions of the system will include a music player that selects music with beats that are on the desired cadence for the day. The feedback from the subjects in this study will also be incorporated in the next version of the system.

Based on the findings of the study, there are early signs of satisfaction from a usability and perceived accuracy point of view, with one exception. The video-based heart rate estimates were perceived as inaccurate in this study. As such, the study findings indicate that there is a need for tools that systematically assess the accuracy of sensory estimates and guide the estimation algorithms accordingly. For example, the algorithms for estimating cadence would be different when the phone is secured on the hip versus when the phone is held by the runner in her or his hand. In these cases, it is the responsibility of the system to employ the correct estimation algorithm by detecting the conditions under which the system is being used. More generally, the study findings suggest that audit mechanisms need to be developed and employed for each estimation algorithm, in order to ensure, verify and quantify the accuracy of its outputs.

One important usability issue to be studied in the future is the motivation of runners to use this and similar mHealth technologies for tracking their runs. There is clearly an interest of (novice) runners to improve cadence for performance gains as is evident from a number of apps that provide feedback through music or otherwise [5,6,7,8,9,10]. However, the evidence for the motivation of using apps for injury prevention is to the best of our knowledge limited. There are several behavioral factors, specific to runners [49], that influence their attitudes towards the level of training and higher risk of injuries. As noted by [50], injury-preventive actions that require behavior modification need to take into account that runners’ perceived susceptibility to sport has multiple predictors, including previous experiences, neuroticism and obsessive passion. Mobile applications for runners thus provide an opportunity to address injury prevention through individualized feedback and various motivational mechanisms, which were out of the scope of this pilot study.

## Figures and Tables

**Table 1:** The demographics of the recruited subjects.

Subject Identifier	Gender	Age (years)	Weight (kg)	Height (cm)
s28ikk	Male	23	67	183
i989kje	Female	24	60	168
w32jbl	Female	24	70	178
b01k1o	Female	25	55	165
p542ok	Male	25	72.5	177
j83bbl	Male	26	72.5	185

**Table 2:** The data types collected about every run.

Data Type	Source of Estimation	Collection Frequency
Energy expenditure	Accelerometer.	Every 60 s.
Cadence	Accelerometer.	Every 15 s.
Speed	GPS.	Every 5 s.
Heart rate	External sensor (chest strap).	Every 1 s.
Heart rate	Video of the runner’s face.	Once before the run and once after the run.
Heart rate	Video of the runner’s index finger.	Once before the run and once after the run.
Distance covered	GPS.	Every 5 s.
Screen light	Android API.	Every 30 s.
Battery consumption	Android API.	Every 60 s.

**Table 3:** Summary of usability reports by the subjects.

Usability Issue	Description
Equipment	Subject b01k1o complained that the heart rate chest strap caused skin irritation. The subject was asked to stop using the strap immediately in order to limit the risk of harm. The strap itself was later tested and no fault in it was found.
Screen light	Data from the screen light sensor shows that subject i989kje regularly checked the app’s interface to view his or her running parameters such as cadence, speed, distance, time and heart rate throughout the run (the screen was turned on more than 80% of the time during subject i989kje’s runs). All other runners ran generally with the screen turned off, only using the haptic vibration and auditory cues as the primary means of feedback (the screen was turned off more than 80% of the time during their runs).
User Experience	Subject i989kje provided a usability feedback in the app regarding the speed and cadence estimates, stating “I stopped a few times during the run and the app did not take it into account”. The data corroborate the feedback by the subject, which seems to have stopped on multiple occasions as can be seen from Figure 4 (run 26). It is worth noting that the app provides an option to pause the current run, which the subject did not use in this instance. Moreover, multiple subjects suggested that speed should be presented in units of mins/mile rather than miles/h.

## References

[1] Van Gent B.R., Siem D.D., van Middelkoop M., van Os T.A., Bierma-Zeinstra S.S., Koes B.B. (2007). Incidence and determinants of lower extremity running injuries in long distance runners: A systematic review. Br. J. Sports Med..

[2] Heiderscheit B.C., Chumanov E.S., Michalski M.P., Wille C.M., Ryan M.B. (2011). Effects of step rate manipulation on joint mechanics during running. Med. Sci. Sports Exerc..

[3] Rowlands A.V., Eston R.G., Tilzey C. (2001). Effect of stride length manipulation on symptoms of exercise-induced muscle damage and the repeated bout effect. J. Sports Sci..

[4] Hamill J., Derrick T.R., Holt K.G. (1995). Shock attenuation and stride frequency during running. Hum. Mov. Sci..

[5] Mapmyfitness. www.mapmyfitness.com.

[6] Runtastic. www.runtastic.com.

[7] micoach. www.Adidas.com/fi/micoach.

[8] Nike+gps. nikerunning.nike.com.

[9] Runkeeper. www.runkeeper.com.

[10] Endomondo. www.endomondo.com.

[11] Aranki D., Balakrishnan U., Sarver H., Serven L., Asuncion C., Du K., Gruis C., Peh G.X., Xiao Y., Bajcsy R. RunningCoach—Cadence Training System for Long-Distance Runners. Proceedings of the 2017 Health-i-Coach—Intelligent Technologies for Coaching in Health.

[12] Kaewkannate K., Kim S. (2016). A comparison of wearable fitness devices. BMC Public Health.

[13] Hussain M., Al-Haiqi A., Zaidan A., Zaidan B., Kiah M.L.M., Anuar N.B., Abdulnabi M. (2015). The landscape of research on smartphone medical apps: Coherent taxonomy, motivations, open challenges and recommendations. Comput. Methods Programs Biomed..

[14] Higgins J.P. (2016). Smartphone applications for patients’ health and fitness. Am. J. Med..

[15] Boratto L., Carta S., Mulas F., Pilloni P. (2017). An e-coaching ecosystem: Design and effectiveness analysis of the engagement of remote coaching on athletes. Pers. Ubiquitous Comput..

[16] Vos S., Janssen M., Goudsmit J., Lauwerijssen C., Brombacher A. (2016). From problem to solution: Developing a personalized smartphone application for recreational runners following a three-step design approach. Procedia Eng..

[17] Lun V., Meeuwisse W., Stergiou P., Stefanyshyn D. (2004). Relation between running injury and static lower limb alignment in recreational runners. Br. J. Sports Med..

[18] Taunton J.E., Ryan M.B., Clement D., McKenzie D.C., Lloyd-Smith D., Zumbo B. (2002). A retrospective case-control analysis of 2002 running injuries. Br. J. Sports Med..

[19] Dixit S., Difiori J.P., Burton M., Mines B. (2007). Management of patellofemoral pain syndrome. Am. Fam. Physician.

[20] Farrokhi S., Keyak J., Powers C. (2011). Individuals with patellofemoral pain exhibit greater patellofemoral joint stress: A finite element analysis study. Osteoarthr. Cartil..

[21] Fredericson M., Powers C.M. (2002). Practical management of patellofemoral pain. Clin. J. Sport Med..

[22] Juhn M.S. (1999). Patellofemoral pain syndrome: A review and guidelines for treatment. Am. Fam. Physician.

[23] Pal S., Besier T.F., Beaupre G.S., Fredericson M., Delp S.L., Gold G.E. (2013). Patellar maltracking is prevalent among patellofemoral pain subjects with patella alta: An upright, weightbearing MRI study. J. Orthop. Res..

[24] Pal S., Draper C.E., Fredericson M., Gold G.E., Delp S.L., Beaupre G.S., Besier T.F. (2011). Patellar maltracking correlates with vastus medialis activation delay in patellofemoral pain patients. Am. J. Sports Med..

[25] Chen Y.J., Scher I., Powers C.M. (2010). Quantification of patellofemoral joint reaction forces during functional activities using a subject-specific three-dimensional model. J. Appl. Biomech..

[26] Reilly D.T., Martens M. (1972). Experimental analysis of the quadriceps muscle force and patello-femoral joint reaction force for various activities. Acta Orthop. Scand..

[27] Lenhart R.L., Thelen D.G., Wille C.M., Chumanov E.S., Heiderscheit B.C. (2014). Increasing running step rate reduces patellofemoral joint forces. Med. Sci. Sports Exerc..

[28] Cavagna G., Mantovani M., Willems P., Musch G. (1997). The resonant step frequency in human running. Pflügers Arch..

[29] Williams K.R., Cavanagh P.R. (1987). Relationship between distance running mechanics, running economy, and performance. J. Appl. Physiol..

[30] Cavanagh P.R., Williams K.R. (1982). The effect of stride length variation on oxygen uptake during distance running. Med. Sci. Sports Exerc..

[31] Verbitsky O., Mizrahi J., Voloshin A., Treiger J., Isakov E. (1998). Shock transmission and fatigue in human running. J. Appl. Biomech..

[32] Candau R., Belli A., Millet G., Georges D., Barbier B., Rouillon J. (1998). Energy cost and running mechanics during a treadmill run to voluntary exhaustion in humans. Eur. J. Appl. Physiol. Occup. Physiol..

[33] Bood R.J., Nijssen M., Van Der Kamp J., Roerdink M. (2013). The power of auditory-motor synchronization in sports: Enhancing running performance by coupling cadence with the right beats. PLoS ONE.

[34] Van Dyck E., Moens B., Buhmann J., Demey M., Coorevits E., Dalla Bella S., Leman M. (2015). Spontaneous entrainment of running cadence to music tempo. Sports Med.-Open.

[35] Johnson C.M., Johnson T.R., Zhang J. (2005). A user-centered framework for redesigning health care interfaces. J. Biomed. Inform..

[36] Abbott P.A., Foster J., de Fatima Marin H., Dykes P.C. (2014). Complexity and the science of implementation in health IT—Knowledge gaps and future visions. Int. J. Med. Inform..

[37] Vedanthan R., Blank E., Tuikong N., Kamano J., Misoi L., Tulienge D., Hutchinson C., Ascheim D.D., Kimaiyo S., Fuster V. (2015). Usability and feasibility of a tablet-based Decision-Support and Integrated Record-keeping (DESIRE) tool in the nurse management of hypertension in rural western Kenya. Int. J. Med. Inform..

[38] Fiordelli M., Diviani N., Schulz P.J. (2013). Mapping mHealth research: A decade of evolution. J. Med. Internet Res..

[39] Aranki D., Kurillo G., Bajcsy R. (2017). Smartphone Based Real-Time Health Monitoring and Intervention. Handbook of Large-Scale Distributed Computing in Smart Healthcare.

[40] Song E., Asuncion C., Balakrishnan U., Sarver H., Serven L. (2016). A Telemonitoring Solution to Long-Distance Running Coaching. Master’s thesis.

[41] Jarv Jarv Run BT Premium Bluetooth Heart Rate Monitor for Android Devices. http://www.jarvmobile.com/productdetail.asp?productid=33499.

[42] Micklewright D., Gibson A.S.C., Gladwell V., Al Salman A. (2017). Development and Validity of the Rating-of-Fatigue Scale. Sports Med..

[43] Borg G.A. (1962). Physical Performance and Perceived Exertion.

[44] Aranki D., Kurillo G., Mani A., Azar P., van Gaalen J., Peng Q., Nigam P., Reddy M.P., Sankavaram S., Wu Q. A Telemonitoring Framework for Android Devices. Proceedings of the 2016 IEEE First International Conference on Connected Health: Applications, Systems and Engineering Technologies (CHASE).

[45] Alshurafa N., Eastwood J.A., Nyamathi S., Liu J.J., Xu W., Ghasemzadeh H., Pourhomayoun M., Sarrafzadeh M. (2015). Improving compliance in remote healthcare systems through smartphone battery optimization. IEEE J. Biomed. Health Inform..

[46] Aranki D., Kurillo G., Yan P., Liebovitz D.M., Bajcsy R. (2016). Real-Time Tele-Monitoring of Patients with Chronic Heart-Failure Using a Smartphone: Lessons Learned. IEEE Trans. Affect. Comput..

[47] Aranki D. (2017). Towards Predictive Medicine – On Remote Monitoring, Privacy and Scientific Bias. PhD thesis.

[48] United States Geological Survey (USGS) EarthExplorer. https://earthexplorer.usgs.gov/.

[49] Johnson R. (1995). Exercise dependence: When runners don’t know when to quit. Sports Med. Arthrosc. Rev..

[50] Stephan Y., Deroche T., Brewer B.W., Caudroit J., Le Scanff C. (2009). Predictors of perceived susceptibility to sport-related injury among competitive runners: The role of previous experience, neuroticism, and passion for running. Appl. Psychol..

